# Spontaneous reattachment of dislocated endothelial graft after non-Descemet stripping automated endothelial keratoplasty: a case report

**DOI:** 10.1186/s12886-018-0684-7

**Published:** 2018-01-22

**Authors:** Aya Kodama-Takahashi, Masahiko Fukuda, Koji Sugioka, Akira Kobayashi, Yoshikazu Shimomura

**Affiliations:** 1 Department of Ophthalmology, Kindai University Faculty of Medicine, 377-2 Ohno-Higashi, Osaka-Sayama City, Osaka 589-8511 Japan; 2 Department of Ophthalmology Graduate School of Medical Science, Kanazawa University, 13-1 Takaramachi, Kanazawa City, Ishikawa 920-8641 Japan

**Keywords:** nDSAEK, Spontaneous reattachment, Dislocated endothelial graft, Corneal edema

## Abstract

**Background:**

Graft detachment is a complication of non**-**Descemet stripping automated endothelial keratoplasty (nDSAEK). We report a case of spontaneous reattachment of an extensively dislocated graft after nDSAEK.

**Case presentation:**

A 54-year-old male underwent penetrating keratoplasty (PKP) for keratoconus in his left eye in 2001. Following graft opacity due to rejection, a second PKP was implemented in May 2014. The graft was kept in good condition after the reoperation and yet, visual acuity (VA) declined due to cataract. PEA+IOL was then performed in May 2015. Because edema appeared in the graft 6 months after the PEA+IOL, nDSAEK was carried out in May 2016. Although the donor graft well attached immediately after the nDSAEK, the graft was almost completely dislocated 3 h later except a temporal part. Air was reinjected into the anterior chamber on the following day and the detachment was resolved. Despite of the treatment, about 1/5 of the graft remained detached and the detachment deteriorated to 3/4 of the graft 9 days later. Because the patient could not decide whether to undergo another operation immediately, we decided to follow him up first and found that the partially detached graft reattached spontaneously 1 month later during the follow-up. Although the cornea had a mild edema remaining in the superior temporal area, his BCVA improved to 1.0. Three months later, the graft remained in position and the cornea kept its transparency.

**Conclusions:**

Spontaneous reattachment was observed during the follow-up in a case that had shown a comparatively extensive graft dislocation after nDSAEK.

## Background

Currently, Descemet stripping automated endothelial keratoplasty (DSAEK) is the most frequently performed surgical treatment for corneal stromal edema due to corneal endothelial dysfunction. In Japan, postoperative complications of cataract surgery or laser iridotomy are the major causes for corneal endothelial dysfunction along with a small number of cases of Fuchs dystrophy. After Kobayashi et al. reported non-Descemet’s stripping automated endothelial keratoplasty (nDSAEK) [1–3], nDSAEK has become the mainstream technique to treat corneal edema in Japan.

Graft dislocation is the most common postoperative complication either after DSAEK or nDSAEK [4]. Graft dislocation is usually treated immediately with air injection into the anterior chamber (AC) or with resurgery to suture the graft to achieve centration. On the other hand, cases of spontaneous reattachment of dislocated donor graft after DSAEK have been reported [5, 6]. We have experienced several cases of nDSAEK graft detachment which did not spontaneously reattach, including cases with extensively dislocated graft and others with persisting folds and wrinkles in the transplanted graft.

Here we report a case of spontaneous reattachment of dislocated graft after nDSAEK that had occurred during follow-up with good visual prognosis.

## Case presentation

**Fig. 1:**
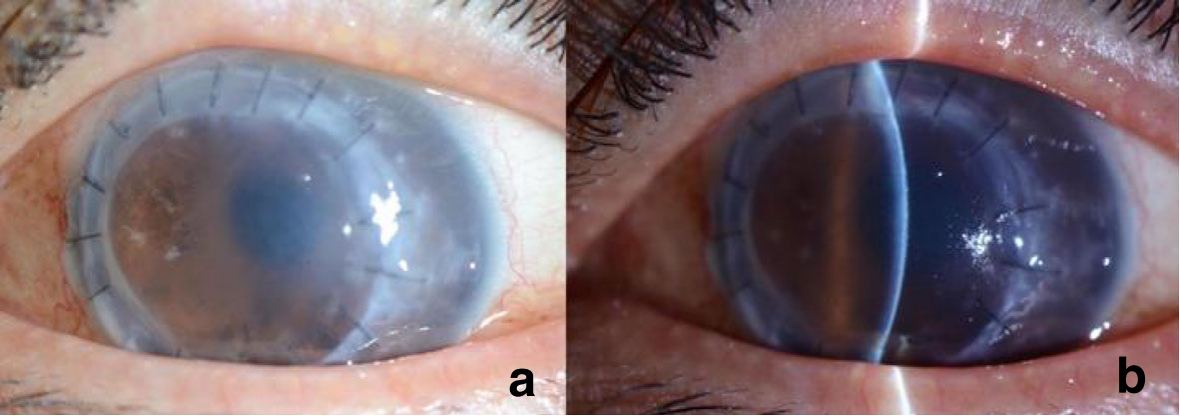
The cornea before nDSAEK. **a** The whole graft showed edema due to corneal endothelial dysfunction after cataract surgery. **b** Corneal stromal edema and corneal opacity were observed

The patient was a 54-year-old male without any systemic diseases. He underwent PKP in 2001 for keratoconus in his left eye, and received another PKP in May 2014 due to opacity caused by graft rejection. After the second PKP, the graft was in good condition but visual acuity declined because of progressed cataract. Subsequently, phacoemulcification with intraocular lens implantation (PEA + IOL) was carried out in May 2015. Six months later, graft edema appeared due to bullous keratopathy [Fig. 1] and the best corrected visual acuity (BCVA) was 0.2.

Under local anesthesia, nDSAEK was performed in May 2016. We prepared a temporal scleral tunnel incision 5.0 mm wide and used a precut donor graft of 8.5 mm. After making four venting incisions, we performed inferior iridectomy with a vitreous cutter. The nDSAEK button was pulled into the AC using a double glide technique [7] with an AC maintainer, a Busin glide spatula (Moria Japan, K.K., Tokyo, Japan), and gripping forceps 23 G (Eye Technology Ltd., Essex, UK). Graft apposition was adjusted and interface fluid between the recipient cornea and the donor graft was expressed through corneal venting incisions. Air was slowly injected into the AC using a syringe equipped with a 30 G needle. However, the air injection could not achieve sufficient tamponade effects. Because the surgery was carried out under local anesthesia and the patient appeared quite stressed and tense, we suspected that this might have caused the high vitreous pressure. Slit lamp microscopy confirmed sufficient graft attachment immediately after the surgery and the operation was completed.

**Fig. 2:**
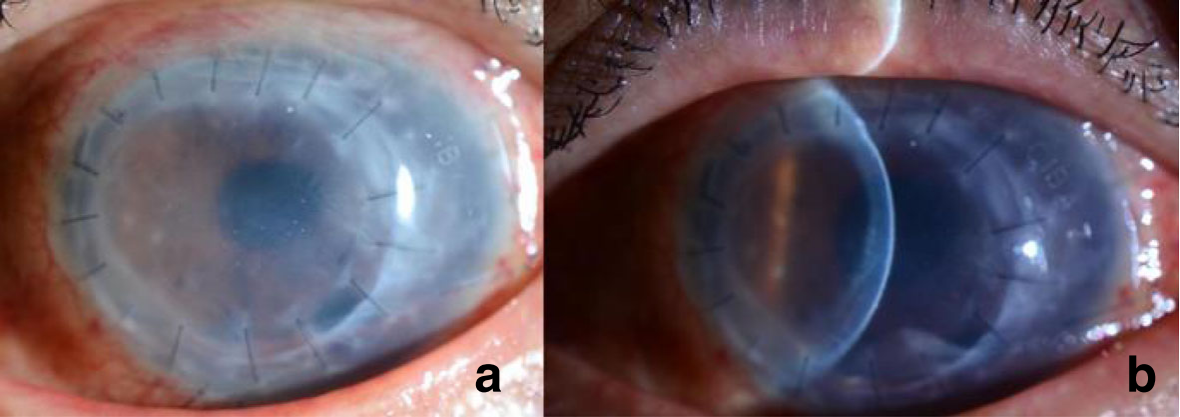
The cornea 9 days after the nDSAEK. **a** Graft dislocation extended to about 3/4 of the whole graft and edema was spotted across the cornea. **b** Edema in the corneal stroma was found and the cornea appeared cloudy

No intraoperative complications were noted. However, the graft almost completely detached 3 h later except a temporal part. Air was reinjected on the following day and the attachment was improved although approximately 1/5 of the graft remained detached in the inferior nasal area. We determined that air reinjection would not further improve the condition and decided to follow him up, which was also the patient’s wish for not having any more operations immediately. Nine days after the nDSAEK, graft dislocation progressed further [Fig. 2]. The area of detachment extended to almost 3/4 of the graft and the BCVA was 0.2.

**Fig. 3:**
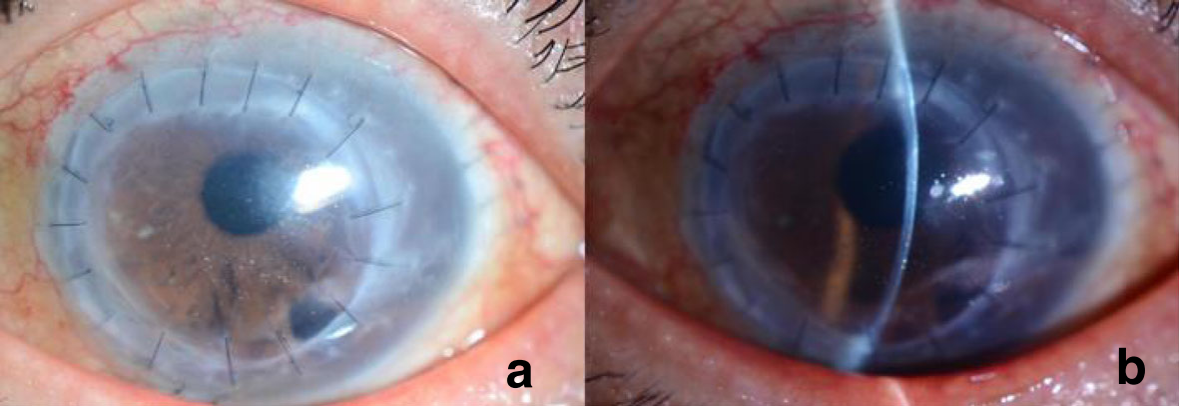
The cornea 1 month after the nDSAEK. **a** Corneal edema was resolved and the whole graft appeared transparent. **b** No graft detachment was noted

**Fig. 4:**
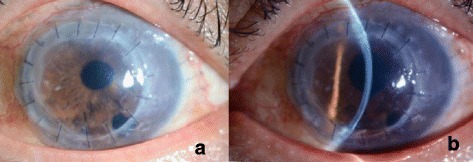
The cornea 3 months after the nDSAEK. **a** The whole graft remained transparent. **b** The graft did not dislocate and the cornea kept transparency

Unexpectedly, spontaneous graft attachment was observed at 1 month after the nDSAEK and the corneal edema had almost subsided leaving only a mild edema in the superior temporal cornea. His vision improved subjectively and objectively, and the BCVA was 1.0 [Fig. 3]. Three months after the nDSAEK, the graft remained in position without any observed complications and the cornea kept its transparency [Fig. 4]. The patient has been followed up ever since.

## Discussion

We experienced a case of a large nDSAEK graft detachment that had spontaneously reattached 1 month after surgery. Our case demonstrated that a close observation without immediate additional operations could be an option for a large but partial graft detachment in the early postoperative period after nDSAEK.

Fuchs corneal dystrophy is the leading cause for corneal endothelial dysfunction in Europe and North America and with Fuchs dystrophy, the recipient Descemet’s membrane and endothelium must be removed to eliminate the guttae that distort vision. In Japan, cataract surgery and argon laser iridotomy are the main factors for corneal endothelial dysfunction and there is no need to strip the Descemet’s membrane. Therefore, nDSAEK has been widely adopted for the treatment of corneal edema. Graft dislocation is a postoperative complication that occasionally occurs after DSAEK and nDSAEK. Reportedly, the decision to remove the Descemet’s membrane or not does not make a remarkable difference in the frequency of postoperative graft dislocation [1–3]. When graft dislocation is observed postoperatively, air injection into the AC, reoperation for graft centration, and graft suturing for insufficient attachment are the common procedures performed immediately to deal with the dislocation. Morishige et al. reported that the duration of bullous keratopathy influences the visual prognosis after DSAEK. They concluded that 1 year after the onset of bullous keratopathy, postoperative visual prognosis becomes poor due to cicatrization [8]. Their finding suggests that early intervention for graft dislocation is necessary to prevent visual loss.

On the other hand, spontaneous graft reattachment has been reported. Hayes and colleagues reported 12 cases of graft dislocation after DSAEK that were followed up without resurgery [6]. In their study, spontaneous reattachment occurred between postoperative 5 days to 7 months. In the 2 cases that required a longer period for reattachment (3 and 7 months), corneal transparency could not be achieved. On the contrary, corneal transparency was achieved in the 2 cases observed with spontaneous reattachment within a shorter period (5 days and 20 days) [6]. Spontaneous graft reattachment does not occur commonly and it is difficult to predict its occurrence. Its mechanism is also unknown. We suspected that the mechanism of spontaneous reattachment may be related to the following: 1) The graft observed with dislocation is always pushed toward the side of the host cornea by the aqueous humor flow and ocular pressure. 2) The aqueous humor between the graft and the host cornea tends to be absorbed by the pumping function of the endothelial cells. Moreover, graft reattachment after DSAEK is considered to share a similar mechanism with the spontaneous reattachment in cases exhibiting detachment of the Descemet’s membrane after cataract surgery, which are considered rare [9].

In the reported case, graft attachment did not appear to have any problem immediately after the nDSAEK, however, graft detachment started 3 h later. We considered 3 possible causes for the graft detachment: 1) The posterior surface of the cornea could have been flattened by the site of the corneal suture since the patient had previously received PKP. 2) The corneal graft of 8.5 mm in diameter might have been relatively too large. 3) Because the operation was performed under local anesthesia and the vitreous pressure was high, the air injection might have been insufficient and caused the fluid to remain between the host cornea and the donor graft.

Likewise, graft detachment could occur in various cases. Although persistent graft detachment can cause poor visual outcome due to corneal fibrosis, the decision between follow-up and immediate reoperations should be made carefully on a case-by-case basis considering the additional burden on patient’s physical condition or possible complications such as iris damage, primary graft failure, and infections.

## Conclusion

In conclusion, our case has demonstrated that even in eyes with a large but partial graft detachment after nDSAEK, the graft detachment can be monitored up to a month for spontaneous reattachment when further intervention appears to be difficult. Although good visual outcomes were obtained in our case, further investigations on the mechanism of spontaneous reattachment, how often it occurs, and in what cases it is most likely to occur will be necessary.

## Abbreviations

AC: Anterior chamber
BCVA: Best corrected visual acuity
nDSAEK: Non-Descemet stripping automated endothelial keratoplasty
PKP: Penetrating keratoplasty
